# A Memoir on the Advantages and Practicability of Dividing the Stricture in Strangulated Hernia

**Published:** 1834-01-01

**Authors:** 


### A MEMOIR ON THE ADVANTAGES AND PRACTICABILITY OF DIVIDING THE STRICTURE IN STRANGULATED HERNIA ON THE OUTSIDE OF THE SAC, WITH CASES AND DRAWINGS. By *C. Aston Key*, Senior Surgeon to Guy's Hospital, &c. &c. 8vo., pp. 161, London, 1833.

MR. KEY is favourably known to the profession, as the author of a work upon lithotomy, as a patron of the straight staff, and as a dexterous surgeon. He is now come forward to advocate a method of operating for strangulated hernia, which though little known, has been less practised, and scarcely at all recommended.

It is generally considered incumbent on those who object to established methods of practice, and propose innovations on ordinary usages, to display the defects of that which they wish to supersede, and contrast with those defects the merits and advantages of the plan they advocate. We are therefore not surprised to observe that the first Chapter in the present work is devoted to observations on the danger attending the common operation for strangulated hernia. The critic has frequently occasion to remark that the natural sentiment of affection for our offspring is not strictly limited to social life, but is felt in the literary and scientific world. That feeling, when carried to a certain and not an uncommon extent, will lead us to depreciate the child of another, in the ratio in which we esteem our own. Hence it is that many who advance new doctrines are found to mete out an inadequate measure of justice to those which they desire to weaken or to overthrow. It will probably appear that Mr. Key may be absolved from this ordinary fault. Yet the opening of the memoir might lead a hasty or censorious individual to surmise, that a tincture of the error is apparent.

"The fatality," says Mr. Key, "that often attends the usual mode of operating for strangulated hernia must have been a subject of deep consideration and regret to every surgeon who bestows much thought on the results of his practice, and who endeavours to make his art subservient to its proper object,—the prevention of suffering and the preservation of life. Oftentimes must he have deplored the necessity of having recourse to an operation that, under some circumstances, holds out so slender a chance of success; and not unfrequently has his duty compelled him to perform it with the conviction that, while it afforded the only hope of relieving the intestine from impending gangrene, it would almost certainly lead to the destruction of the patient.

That this is not an exaggerated feeling of apprehension as to the dangers attending the operation for strangulated hernia, the records of large hospitals and the memoranda of private practice, if candidly stated, will abundantly testify; and it cannot but strike the reader of the periodical publications, that they alone furnish sufficient evidence of its mortality, in the numerous *post-mortem* examinations, compared with the number of successful cases detailed in these works." 2.

The candid surgeon will be tempted to inquire, whether the operation for strangulated hernia does really deserve so unfavourable a character. We apprehend that the general opinion is somewhat adverse to that of Mr. Key. His accusation appears to be based on two leading circumstances—first, that the unsuccessful operations are numerous, at least if we may judge by the periodical publications; and, secondly, that the operation is serious in itself, and materially adds to the dangers of the patient.

It may be stated in reply to the first observation, that the nature of modern periodical literature would rather tend to the publishing of fatal than of fortunate results. The weekly journals are chiefly fed with the cases occurring in the hospitals. The patients taken to those institutions, are necessarily such as present the most unfavourable circumstances to the operator. Urgent and protracted efforts have usually been made to reduce the tumor by the taxis, and the operation has been commonly delayed to a very prejudicial extent. We may add that the worst of the hospital cases are commonly selected for the public, the reporters considering that the majority of those which are successful present no peculiar features of interest.

It may safely be questioned if the second branch of Mr. Key's accusation, is consistent with the experience of the mass of surgeons. We have heard a very eminent operator declare, that he believed the operation to be scarcely attended in itself with danger. So widely has a similar opinion prevailed, that the knife is now resorted to, almost universally, at a much earlier period than was formerly selected. The example of French surgeons has frequently been quoted as illustrative of the safety of an early operation. If necessity or timidity conspire to protract the employment of this decisive measure, it may then be allowed that the chance of recovery from the common operation is alarmingly diminished. But reason instructs us that, under such circumstances, the danger of the particular method is not magnified, but the chance of benefit from any is diminished.

Whether we are inclined to allow or to dispute the estimate of Mr. Key, it must be owned that the argument resolves itself to this—is the old plan or the new attended with *least* risk? The most enthusiastic advocates of the former must admit, that often it is not successful, and that sometimes it may not be safe. The question becomes one of comparative value, and to judge of this we must listen to the pleadings. The third, fourth, and fifth divisions of the volume may be said to contain the feelings and the reasonings of Mr. Key upon the subject, and we turn to them at once, without stopping to notice an able and interesting historical sketch of what we may improperly denominate the new operation.

We proceed to consider Mr. Key's enumeration of the advantages of the reduction of the hernia, by division of the stricture without opening the sac. These advantages may be enumerated in the following order.

1. "A prominent character of the operation, and one that raises it above many of the objections that have been brought against it, is that should the attempt to execute it fail, either from want of dexterity on the part of the operator, or from any peculiar difficulty in the case, the operation can be completed in the ordinary way, by laying the sac open. A surgeon may possibly find great and insuperable difficulty in dividing the stricture externally to the sac; or, having divided the stricture, he may be unable, by the best directed efforts, to return the contents of the hernial tumour: in such a case, he has not brought himself into any dilemma by his unsuccessful attempt; the operation may proceed, as if it had not been made; and neither patient nor surgeon are in a worse position than if the sac had been opened in the first instance, without the attempt to preserve it entire. It is no slight recommendation of the operation, that its failure involves the surgeon in no embarrassment, but leaves him at liberty to adopt the old mode of operation." 48.

2. There is a modification that the surgeon may resort to. He may make an opening in the sac below the stricture, introduce a director, convey this under the stricture, and divide it. Mr. Key observes that no objection can be urged to the proceeding in a case of strangulated recent enterocele. But he likewise remarks, that the only instances in which it can be decidedly advantageous or necessary, are those in which there is an unusual degree of tightness in the stricture at the inner ring in inguinal hernia, rendering it impossible to divide the tendon without wounding the neck of the sac; or, a thickening of the neck of the sac in the femoral hernia.

3. Sir Astley Cooper's authority is quoted, and it seems very favourable to the proposal of Mr. Key. The former excellent and able surgeon expresses his conviction, that the operation will be found, if performed early, to be free from danger, and not be accompanied with difficulty. He observes that by this means we avoid the danger of wounding the bowel, and that if the epigastric artery should be cut, it cannot bleed into the abdominal cavity. He recommends the operation in herniæ of great size, because the viscera will not be exposed to being handled, and can subsequently be more readily retained in the abdomen. He advises that the sac should not be opened in large irreducible herniæ, as the separation of extensive adhesions would expose the intestine to great risk of inflammation.

4. "I need not again urge the main benefit derived from the external division of the stricture in the non-exposure of the patient to those causes of inflammation, to which the ordinary operation subjects him, and, as experience frequently proves, with most unhappy consequences. The exposure of a bowel in a state of incipient or active inflammation, the handling it in this susceptible state, the incision made into a peritoneal bag already disposed to, if not in an actual state of, inflammation, are, as every surgeon will admit, and as his forcible efforts to reduce the hernia without the knife prove that he feels them to be, dangers of no ordinary magnitude to a patient labouring under a strangulated intestine. I do not feel that I have exaggerated the risk of inflammation; for frequently enteritis comes on, when at the time of the operation the bowel appears to be healthy, and the abdomen free from tenderness; and when general inflammation precedes the operation, the release of the intestine by the knife rarely succeeds in checking it." 51.

5. Mr. Key alludes to a circumstance that sometimes occurs. The patient appears to do well after the operation, but in two or three days he begins to sink, and after several more he dies. The cause of death is found in the dark colour and lacerable condition of the strangulated portion of bowel, and the vascular state of the surrounding parts. Such a case seems to mark the enfeebled powers of the constitution, and Mr. Key conceives that the exposure of the bowel that resists inflammation and destruction so faintly, can scarcely be considered as other than injurious.

6. In cases, says Mr. Key, in which great depression of the powers are observed to precede the operation, death sometimes rapidly takes place without any other obvious cause than the exposure of the bowel. The condition of the patient is often found to be manifestly worse after the operation, and stimulants are obliged to be plentifully administered, in order to sustain the sinking powers of life. This may happen without inflammation of the abdominal cavity, or gangrene of the bowel; and is attributable solely to the depressing effect of the operation. The pulse, which before the operation was feeble, becomes fluttering, and scarcely perceptible; the countenance, which was anxious, now bespeaks the approach of death; the skin is covered with a clammy moisture, and the whole frame is seized with a restlessness that gradually ends in the calmness of dissolution." 54.

7. Mr. Key employs as an analogical argument, the injurious effects that are known to succeed to contused wounds. He compares the condition of the exposed intestine to that of the tissues beneath the skin, submitted to the pernicious influence of the atmosphere, after a contusion and wound of the integument. He pushes this argument to a point which could only be perfectly justified, by a long experience in the new operation, an experience which it has not hitherto received. He observes, that if the contusion be not accompanied by a breach of the surface, no harm is anticipated; and just so if the bruised contents of a hernia are returned without a wound of the peritoneal sac, and consequent exposure, inflammation, if it does come on, seldom proves severe, and still more rarely fatal.

8. An eighth consideration is founded on the smaller liability to the production of hæmorrhage, presented by the new than the common operation. Independent of the circumstance, that, the sac being unopened, the blood, if it flows, will not enter the cavity of the abdomen, the vessels are much less exposed to danger. The director, says Mr. Key, in both inguinal and femoral hernia is so placed, that the knife is carried before the vessels; and thus the epigastric artery, and the obturator, when it crosses the femoral sac, will be more likely to escape. The cremaster branch of the epigastric, which, in one case mentioned by Mr. Lawrence, furnished a very copious bleeding, is out of the reach of the knife when the latter is passed on the outside of the sac.

9. The intestine is likewise placed beyond the reach of danger from the knife. It is occasionally wounded in the ordinary operation.

After the division of the stricture it occasionally happens, that in drawing down a further portion of the bowel, that part of it formerly constricted gives way. This occurrence is more frequent, for an obvious reason, in femoral hernia than in inguinal. Such an accident cannot attend the division of the stricture on the outside of the sac.

10. "Small collections of pus at the mouth of the sac, attended with intestinal irritation and peritoneal inflammation, are occasionally found, on inspection, after the operation of opening the sac. The following case presented this appearance, together with a change in the condition of the mucous follicles of the intestine. I will not aver that this will not happen if the sac be left entire; but it is probable that the latter mode of proceeding would much diminish the chance of suppuration. The intestine was much chilled before it was returned: this frequent and fruitful source of subsequent reaction and inflammation would be wholly avoided." 70.

We need not relate the case.

The foregoing enumeration comprises the advantages attributed by Mr. Key to the operation he proposes. We have placed them before the reader in a more methodic form than that which they wear in their native volume. A critical review would readily demonstrate, that some of the arguments employed by Mr. Key are possessed of more force than others, and perhaps it might be shewn, that a few are not only inconclusive, but hostile to the cause for whose support they are adduced. It is doubtful whether those which we have numbered as the fifth, the ninth, and the tenth, are really calculated to furnish the assistance our able author would appear to expect from them.

The candour of Mr. Key will spare us the ungrateful and the unsatisfactory office of urging objections against the operation. They are fairly stated by himself, and a patient analysis will readily oppose them to the list of recommendations and advantages to which we were just referring. Petit has put on record some of the objections, accompanied with the antidote of his own reply. We will endeavour to select and methodize these objections, in the manner we have adopted in considering the favourable side of the question.

1. It is urged that the new operation is difficult. To this M. Petit replies, that the usefulness and practicability are the points to be decided. He also denies that great difficulty exists, when a properly-adapted director is chosen.

2. It is argued that the fluids shut in the sac, being returned into the abdomen, may irritate and inflame the peritoneum. Cheselden observes, in his Anatomy, that he found above two pounds of fetid matter in a hernial sac. But the natural and obvious rejoinder of M. Petit is conclusive. We always endeavour to reduce a hernia by the taxis, without permitting a dread of the irritating quality of the fluid to prevent us. Experience proves that the fear and the objection are equally idle.

3. Heister remarks that the prolapsed omentum or intestine is sometimes in a state of suppuration, which cannot be discovered if the sac remains entire.

4. The same author observes, that the omitting to open the sac may easily occasion a return of the disorder. It is greatly to be doubted if such a division does really tend to prevent that effect.

5. The operation is not adapted to those inguinal ruptures in which the peritoneum is already lacerated. The advocate may admit this scholastic objection, without any material disadvantage to his case.

6. Richter observes, that the operation is possessed of no advantage above the ordinary method, and that the principal argument in favour of it, rests on the *imaginary* danger of opening the hernial sac. He remarks that it is immaterial whether the latter be divided or not, and that a radical cure is less likely to follow if no division has been made.

7. When the stricture is composed of the neck of the sac, the new operation would be out of the question. Mr. Key believes such a source of constriction to be of very rare occurrence.

8. But perhaps the most important reasonings are founded on the existence of adhesions in the sac, and the gangrenous condition occasionally shewn by the intestine. And first of the presence of adhesions.

Heister has placed this among his objections—that adhesions of the intestine or omentum may occur, which cannot be separated without opening the sac. Richter, on the other hand, observes, that the operation is really indicated, when the intestines adhere to each other and to the sac, because the latter can scarcely be opened without the risk of injuring its contents. Here, where he allows an indication, he denies the practicability of the plan, because the hernia cannot be reduced without the prior exposure of the intestines, and destruction of the adhesions that impede their functions.

Mr. Key replies at some length to this objection.

"Adhesion of the intestine may possibly constitute a case that will not admit of it being returned without opening the sac. Soon after an intestine becomes incarcerated and its circulation impeded, effusion of fluid takes place, preventing the bowel coming in contact with the parietes of the sac. If, however, inflammation takes place in the peritoneal covering of the bowel soon after its descent, the effusion is of a plastic kind, and adhesion ensues between the opposed surfaces. The presence of fluid in the sac will in most cases be sufficient to determine the free condition of the bowel, or the slight nature of the adhesion; while its absence, especially if the tumour be hard and tender to the touch, may lead to the suspicion (not to the certainty) of adhesion having taken place. When, under such circumstances, the sac is opened and the bowel exposed, a very slight degree of force is required to separate the adherent intestine. It is true that this separation is not effected by the pressure which the tumour undergoes in the use of the taxis ; but if the stricture be divided, and the intestine be free to pass into the abdomen, the adhesions, consisting of a thin pellicle of fibrine of perhaps a few hours' formation, will probably give way as soon as pressure is made upon the tumour. This, however, is only opinion ; experience alone can decide it. But should the intestine resist the attempt of the surgeon to disengage it, he has then the alternative of opening the sac and concluding the operation in the usual manner." 94.

Pursuing the train of reasoning contained in this quotation, Mr. Key concludes that recent and slight adhesions of the intestine are not matters of such moment as lightly to intimidate the surgeon. He believes that many of the cases, in which the fatal result has by some been attributed to the presence of such adhesions, have really been instances of death from peritoneal inflammation, arising, perhaps, from the exposure of the intestine. He allows that such adhesions as confine the gut at a very acute angle, must check the progress of its fæcal contents, but contends that such cases are far from common. He admits that adhesion of the bowel at the orifice of the sac would require the exposure of its contents, in order to free their baneful attachment. He seems to conclude, at all events he hints, that the gut could not be reduced in such cases without difficulty, and wherever an unusual amount is experienced, he recommends the sac to be instantly opened, that the operator may determine the nature of the resistance.

" In irreducible intestinal herniæ a difference of opinion may exist, whether it would be advisable to remove the cause of obstruction without opening the sac. When symptoms of strangulation come on in irreducible herniæ, they are found most commonly to depend upon the mechanical obstruction to the passage of the fæces, rather than to any actual strangulation of the vessels of the intestine; when the sac is opened and the bowel exposed, it does not present the usual venous hue of extreme congestion, but rather a florid colour, indicating an increased activity of circulation, or the first stage of inflammation. That any advantage can attend the opening of the sac, and the exposure of a bowel in this condition, appears to me very questionable. Whatever relief is to be afforded to the distressed bowel can as well be given by dividing the stricture on the outside of the sac, and thus enlarging the opening of communication with the abdomen. I believe that this mode of freeing the bowel from pressure, and the administration of a dose of calomel and opium to tranquillise for a time the action of the bowel, followed in a few hours by a mild purgative, would be sufficient to restore the function of the intestine and to remove the symptoms of obstruction. The opening of the sac, with a view of removing the adhesions, is an operation, to say the least, hazardous in its consequences, when the division of the adhesions can be effected, and oftentimes useless, as the intestine in old herniæ sometimes adheres so extensively to the parietes of the sac, that it is impossible to detach them without endangering the bowel; and the practice of returning the intestine with portions of the adherent sac, does not appear to possess any decided advantage, as the cut surface of the sac will, in all probability, contract fresh adhesions to the parts with which it may come in contact." 100.

Further on, Mr. Key remarks—

" When my attention was first directed to this subject an adherent omentum seemed to me likely to throw some difficulty in the way of reducing a portion of intestine, that might have descended into the sac and have become strangulated. The difficulty of applying direct pressure to a piece of intestine so circumstanced, and the possibility of it being entangled by the omentum, appeared to me rather to forbid the operation, and to require the sac to be opened. But the very first case in which I attempted to perform it, was an old irreducible umbilical hernia, in which a fold of intestine had recently descended; and to my surprise, instead of finding any difficulty in returning it, the edge of the tendon being divided, very slight pressure made on the sac immediately caused the bowel to return. And I believe that, in many of those cases in which the intestine is apparently entangled by the omentum, a slight division of the stricture would be sufficient to release the bowel and induce it to return; for although, when the sac is opened from the outside, the omentum may seem to entangle and to detain the bowel in the sac, yet in reality the channel from the abdomen by which the intestine has descended may be quite direct, and the pressure of the omentum on the intestine may be only apparent, and may cease as soon as the tendon is divided. The adhesion of the omentum to the neck of the sac will not add to the difficulty of dividing the stricture." 102.

In some cases, the operation has disclosed the omentum in such a condition, that its removal by the knife has been advised and practised. Mr. Key observes that, when the sac is left entire, a proceeding of this nature would probably be unnecessary, as well as impracticable.

9. It is allowed by all, that a gangrenous condition of the contents of the sac requires its division, and the free exposure of the parts. The aim of Mr. Key is, therefore, to discover some means of determining the existence of this state. The ordinary symptoms we need not point out. They consist of the state of the tumour, and the general features of the case. Mr. Key has witnessed a case in which the gangrene of the gut was marked by a peculiarly fetid smell, perceptible during the progress of the operation. But, independently of the ordinary marks of gangrene, and this which he has once observed, Mr. Key offers no additional means of detecting this formidable state. The symptoms to which we have alluded are probably only observed when mortification *has* ensued. The formidable and not unfrequent class in which the intestine is *becoming* gangrenous, present no feature by which they can be with any certainty distinguished. This circumstance must exercise a considerable influence on the mind of the profession. When the surgeon returns the gut without opening the sac, he feels that, in many cases, he cannot be assured that it is not on the verge of sphacelus.

It occasionally happens that the intestine sloughs after it is replaced in the abdomen, and the fæces pass for a time through the wound. Were the sac unopened those fæces would probably collect in the cavity of the peritoneum, and materially aggravate the danger of the patient. Mr. Key anticipates that the non-exposure of the gut would tend so greatly to prevent this occurrence, that the balance of advantage would incline to the side of the operation. But the candid enquirer may perhaps suspect that the benefit and prevention are only conjectural, and the risk positive and certain. Those who are convinced by the confidence of the reasoner may be more inclined to agree with Mr. Key, when they find him so satisfied with the justice of his expectation, as to declare, that the operation of leaving the sac entire appears to him peculiarly applicable to cases of this description. He considers that a full examination of the subject should diminish the apprehension of danger from a gangrenous state of the intestine.

10. This objection is brought forward by Mr. Key himself.

"From the difficulty which occasionally attends the return of a large mass of omentum, it is not improbable that the sac of an omental hernia will often require to be opened, when the descended portion is voluminous and the inguinal canal narrow. Even when the abdominal rings do not produce pressure sufficient to cause strangulation, it is often very difficult to return by the taxis a mass of incarcerated omentum after a descent of only a few hours. The return of an enterocele is readily effected from the regular and smooth surface of the intestine; but the lobular form and irregularity of the omentum require a more prolonged manipulation, and allow of it being reduced as it were only piecemeal: unless the rings are very wide, it does not slip up at once, and not unfrequently requires some days before the whole mass can be completely reduced. Some years ago I experienced so much difficulty in returning it after dividing the stricture, that I was obliged to open the sac. This case I have occasion to refer to when describing the operation for inguinal hernia. Should a portion of intestine have descended behind the omentum, the former might be returned, first, by making pressure on the back part of the swelling, and this would facilitate the return of the omentum. After the stricture is divided, the intestine would readily recede if pressure be made directly upon it, and more space would be afforded for the reduction of the omental mass." 119.

We believe we have offered a very candid analysis of the arguments for and against the operation, contained in the work of Mr. Key. Reviewing has been termed the ungentle craft; analysis may fairly be styled the unpleasant one. Those who are unused to literary composition, display, when they pass from professional pursuits to publish their views or their experience, an unaptness in reasoning and a ruggedness in style, that render the function of critical digestion a work of pain and of difficulty. We have found some trouble in placing in connected order the reasons arranged in the preceding pages. Did we venture to indulge in the vanity of advice, we would hint to Mr. Key, that in a second edition of this valuable work, he might condescend to imitate the critic in the homely though useful virtue of precision.

It fortunately does not fall to the province of the analytical reviewer to balance conflicting evidence, and assume the power of pronouncing judgment. He acts as a public cook, and prepares in a palatable form, the viands which his patrons and employers taste.

Yet we think that we shall not exceed our duty, if we venture to display the essential points on which the controversy rests. The advocate of the new operation, objects to the old that it is fraught with danger, and that this essentially depends on the division of the sac and exposure of its contents to the air and to the gross manipulation of the surgeon. The supporter of the ordinary operation replies, that the opening of the sac does not contribute to aggravate the risk, and constitutes only an imaginary peril.* He observes that in many cases it is owned by all that the contents of the sac must be exposed—that it is frequently a matter of great difficulty to distinguish the circumstances that require the division of the sac—that intestine may be indiscreetly returned into the abdomen in a state approaching to gangrene, or bound in such a manner by adhesion to itself or the omentum, as not to be capable of exercising its function after its reduction—that if any mischief subsequently ensues, there is not that ready vent for the escape of pus, of fæces, or of slough, which exists when the sac has been divided—and finally that we are voluntarily acting in the dark, and depriving ourselves of an important criterion of the condition of the parts, and a valuable guide in our subsequent proceedings.

The intelligent reader will probably disregard the *number* of the arguments against the operation, and attach a just degree of importance to the general bearing of the reasoning for it. Yet we doubt if all surgeons will freely admit the danger of opening the hernial sac, and some perhaps will accuse Mr. Key of greatly over-rating it. We suspect that the non-division of the sac will never be very extensively practised, nor become the common mode of operation. A prejudice will always lurk in the mind in favour of ascertaining the condition of the gut or the omentum, and the operator who has proceeded as far as the sac, will in general believe that caution and prudence urge him to open it. At the same time the facts and the reasonings of Mr. Key must exercise an extensive and permanent influence, and in many cases the surgeon will be led by the presence of favourable circumstances, to perform the operation he advises. Such is the anticipation we would venture to indulge from a careful consideration of the question.

Six cases are related by our able author; two were attended with a fatal result. One of the more fortunate was an instance of severe umbilical her-

* Richter, as quoted by Mr. Key.

nia, and highly illustrative of the value of the operation. We will briefly notice two of the successful cases.

*Case* 1. Mrs. J. æt. 59, consulted Mr. Key for a femoral hernia, which he had some difficulty in reducing, and for which he recommended the employment of a truss. The advice was not adopted, but the hernia did not reappear. Two years after this, on the 17th December, 1832, he was summoned to attend her, in consequence of the tumor having descended in the water-closet. Mr. Key tried the taxis for twenty minutes unsuccessfully. Ice was then applied till the evening, and the taxis repeated, with no better result. Some Dover's powder was given her at bed-time, and in the morning a castor oil enema was ordered. In the course of the night she had vomited several times, and the abdomen was becoming more tender. The castor oil had not brought away any fæculent matter. Mr. Key had now recourse to the operation without any further delay.

"With the assistance of my dresser, Mr. Langley, I exposed by means of a crucial incision the fascia propria of the tumour, and, making an opening into it so as to expose the fatty investment of the sac, I endeavoured to pass the director towards the stricture; but, owing to the angle formed by the tumour with Poupart's ligament, I was obliged to divide the fascia more freely towards the neck of the sac, in order to reach the seat of stricture. The director was then passed without difficulty between the fatty covering of the sac and the outer layer of the fascia propria, and carried under the stricture. The blade of the bistoury was then passed along the groove, and the stricture divided in a direction towards the umbilicus. The intestine was immediately released from pressure; for, by applying a very moderate force to the tumour, the intestine immediately slipped up; and a small piece of omentum that remained, was readily returned by a little farther manipulation." 137.

The bowels were freely and spontaneously relieved after the operation. On the 22d there was a slight disposition to diarrhœa, and a blush of erysipelas appeared about the wound. This, however, subsided, the wound did well, and on the 8th of January a truss was applied.

*Case* 2. This was seen by Mr. Key in company with Mr. Désormeaux, of Pentonville. The commencement of the case is given in the words of Mr. D.

"Mrs. T., about fifty years of age, sent for me on Saturday, the 12th of January, on account of a femoral hernia, which she stated to have existed for several years, but which she had been able to keep up with a common truss. It had occasionally descended, in consequence of the truss not fitting her. In making some exertion, it suddenly came down this afternoon, but felt, as usual, when it happened to make its appearance, soft and free from pain. In a short time it became much harder and painful, producing a great sense of tightness across the belly, with nausea, which soon ended in vomiting. I saw her in the evening, and found her suffering great pain with the hardest hernia I had ever felt. The tumour seemed to be filled with a mass of hard indigested substances, which could be felt through the skin, the coverings being very thin. I attempted to reduce it by the taxis, which was steadily kept up for twenty minutes, and then she tried to reduce it in her usual way, but without success. It appeared to me impossible to reduce the tumour, without first kneading the solid contents of the bowel through the femoral ring, which, from the great solidity of the matter, appeared to me almost hopeless. I took from the arm as much blood as brought on a fit of fainting, and then made a second attempt to reduce it. Conceiving that all measures would be useless while the contents could not be moved, I proposed having further advice." 139.

On the following morning Mr. Key examined the lady. The tumour was remarkably hard and very tender, about the size of a small orange, and accompanied with urgent symptoms. Mr. Key tried the taxis in vain, and then proceeded to the operation. The stricture was distinctly heard to give way, and the contents of the sac were immediately reduced as soon as pressure was attempted.

The sickness ceased, but the bowels were not opened till the evening, and that with the assistance of a draught containing the sulphate and the carbonate of magnesia. The night of the 14th was bad, and much pain in the abdomen, with a sense of tightness, were complained of on the following day. The pulse was 110. Leeches were applied and aperient draughts continued. In the evening she was ordered a dose of Dover's powder. The wound healed by the first intention, and on the 18th she was convalescent.

It only remains to notice Mr. Key's particular directions for operating on the three varieties of hernia. And first of that of the inguinal kind.

The following remarks on the method of operating are not undeserving of insertion; they do not admit of satisfactory abridgment.

"The extent, as well as the form of the incision through the integuments, may seem of minor importance, except as far as it tends to facilitate the after steps of the operation; yet it may be as well to disturb the subjacent cellular membrane as little as possible, as inflammation is less likely to follow, and to assume the form of erisypelas. For this reason, the inverted T incision, usual in the operation for femoral hernia, may be in most cases reduced to a single incision, either at right angles to Poupart's ligament, or in a transverse direction across the tumour. In patients who are spare, and in whom the neck of the sac lies at no great depth from the surface, it is unnecessary to disturb the cellular membrane by turning aside the flaps of the integument. This will diminish the suppurative inflammation, and in such cases will afford ample room for the operation. I have not made trial of the perpendicular form of incision, but a single transverse one I have found sufficient, when the integuments have been loose, and the tumour not large. The superficial fascia adheres firmly to the common integuments, and is usually turned aside with them, especially when the latter are pinched up for the purpose of making the first incision. The fascia propria is, therefore, quickly exposed, and forms the first distinct covering of the tumour, being darker than the more superficial cellular investment. It is under the outer layer of this fascia that the adipose structure is formed, and which often assumes the appearance of omentum. The director easily makes its way under this fatty matter as far as the neck of the sac, which lies deeper than the operator at first supposes. The point of the director should be applied rather to the inner than to the outer part of the neck of the sac, as it will be found more easily to pass under the stricture at this part. It should not at first be attempted to be thrust under the stricture, as the firmness of the parts forming the stricture would resist it. But the seat of stricture being felt, the operator should depress the end of the director upon the sac, which will yield before it, and then, by an onward movement, the director slides under the stricture. The usual seat of stricture in a femoral hernia is too familiar to need any elaborate description."

"The band that produces the constriction at the femoral aperture is not entirely a process from Poupart's ligament, but is also formed by a tendinous band on the fore part of the femoral sheath, where the fascia transversalis passes in a funnel form behind Gimbernat's ligament, to be inserted into the pubis. The thin tendinous border that descends backwards from Poupart's ligament is attached to the front part of the sheath of the femoral vessels ; and any attempt to push a director under this thin border, anteriorly to the sheath, would meet with great resistance ; and, if it were successful, its division would not sufficiently liberate the sac from pressure." 145.

In inguinal hernia the incision should be made higher than it is in the common operation. Commencing at the neck of the tumor it should be carried downwards for an inch and a half. The tendon of the external oblique, where it forms the ring, being exposed, a small opening should be made in it just above the ring, to admit the end of the director, and enable the operator to distinguish if the opening be at the upper or the lower opening. If the stricture is seated at the lower ring, the director must be passed under its margin, and its division must be carried to a sufficient extent.

"If the stricture exists higher up at the neck of the sac, where it will be found in the majority of herniæ of this description, the opening in the tendon should be enlarged to the extent shown in the second drawing, for the purpose of passing the director under the deeper stricture. The lower margin of the two muscles will be brought into view, with some of the descending fibres of the cremaster. These may be separated by disturbing the cellular membrane with the end of the director ; and the instrument may then be introduced under the transversalis muscle till it reaches the stricture. In the subject, the director, when introduced in this manner, passes before the tranversalis fascia ; this will diminish what little risk there may be of wounding the peritoneum, and will carry the knife further from the epigastric artery ; the tenuity, however, of this fascia, will, perhaps, often allow the director to pass beneath it. The instrument should be depressed upon the sac, in order to carry its point under the border of the transversalis, which may be divided to the extent required. This operation is more difficult than the division of the stricture in femoral hernia ; the principal difficulty lies in the accurate separation of the lower edge of the internal oblique muscle, for the easy passage of the director. Drawing II. represents the parts, with the instrument passed under its edge. The stricture, however, is not so firm in inguinal as in femoral hernia, and the introduction of the director under the transversalis tendon will not be difficult, where it is fairly passed up to the neck of the sac before the attempt is made. The steps of the operation will be much the same in those smaller hernia, which are lodged in the inguinal canal. When the stricture is divided, a greater degree of pressure will be required to return the contents of a large inguinal hernia, on account of the distance from the neck of the sac to the bottom of the tumour, and especially when the omentum forms a part of its contents." 149.

In small bubonocele, in which the protrusion has scarcely reached the external ring, the same manner of operating may be followed.

Umbilical hernia is, according to our author, the one that most requires the sac to be left entire. The operation for this hernia is too often unsuccessful.

The division of the tendinous margin of the umbilical aperture is not difficult ; it requires care, on account of the extreme thinness of the sac ; and the operation, therefore, consists in a cautious exposure of the linea alba, where the tumour emerges from the abdomen. The orifice of the sac is rendered readily accessible at its upper part by the descent of the swelling towards the pubes ; the sac, when it emerges from the abdomen, does not extend equally in all directions, but gradually makes its way downwards, in consequence of the weight of its contents; and therefore, in old large herniæ, though the aperture in the tendon bears but a small comparison to the size of the tumour, it is scarcely at all overlapped by it at the upper part.

A case is related by our author in which the operation succeeded à merveille. We regret that we cannot insert it.

We conclude our notice of the present work. We believe we have displayed in an impartial manner the views of Mr. Key. Fortunately the expression of a critical opinion is neither desirable nor necessary, the question being one which experiment and experience alone can decide. Mr. Key may complain that we have shewn no leaning to his side. Prepossession perhaps is engaged against him, but prepossession will give way to the influence of truth. Should Mr. Key's sentiments prove to be correct, their triumph in spite of opposition and coldness will be equally certain and more satisfactory. We have merely to remark that Mr. Key deserves considerable credit for the pains which he has taken to bring the operation before the profession. The manner in which he has performed his task is highly deserving of praise.

